# Multimeric, multivalent fusion carrier proteins for site-selective glycoconjugate vaccines simultaneously targeting *Staphylococcus aureus* and *Pseudomonas aeruginosa*†

**DOI:** 10.1039/d4sc08622h

**Published:** 2025-02-20

**Authors:** Charlotte Sorieul, Bartal Mikladal, Dung-Yeh Wu, Barbara Brogioni, Cinzia Giovani, Giusy Adamo, Giacomo Romagnoli, Immaculada Margarit Y Ros, Jeroen Codée, Maria R. Romano, Filippo Carboni, Roberto Adamo

**Affiliations:** a Leiden Institute of Chemistry, Leiden University Einsteinweg 55 2333 CC Leiden Netherlands; b GSK Siena Via Fiorentina, 1 53100 Siena SI Italy roberto.x.adamo@gsk.com filippo.x.carboni@gsk.com

## Abstract

*Staphylococcus aureus* and *Pseudomonas aeruginosa* are major antimicrobial-resistant pathogens that often synergize in polymicrobial infections, such as chronic wound infections. These notorious and increasingly resistant bacteria contribute significantly to reduced antibiotic efficacy. Despite their substantial clinical burden, the urgent need to combat bacterial resistance and extensive research efforts, no vaccines currently exist for either bacterium. Glycoconjugate vaccines, which extend the range of suitable vaccine antigens to bacterial carbohydrates, could play a major role in this emergence. This study introduces a multiepitope vaccine conjugating *S. aureus* capsular polysaccharide serotype 8 to a chimeric protein fusing Hla and PcrV, two potent cytotoxins from *S. aureus* and *P. aeruginosa*, respectively. A conjugation strategy based on selective targeting of a purposefully introduced histidine tag was developed to preserve the structure and antigenicity of epitopes from the two proteins, leveraging their dual role as a carrier and antigen. This multivalent, multimeric and multipathogen construct successfully elicited antibodies against all three antigens as well as functional protection. This proof-of-concept highlights the potential for advanced vaccines targeting polymicrobial infections and bacteria with complex pathogenesis calling for multivalent formulations. It also points out the power of site-selective conjugation as a tool for vaccine manufacturing.

## Abstract 2

A 3-in-1 glycoconjugate vaccine, incorporating multiple copies of three antigens from *Staphylococcus aureus* and *Pseudomonas aeruginosa* into one construct, was generated, leveraging a histidine-directed conjugation, and elicited a strong immune response against each antigen in mice.

## Graphical abstract

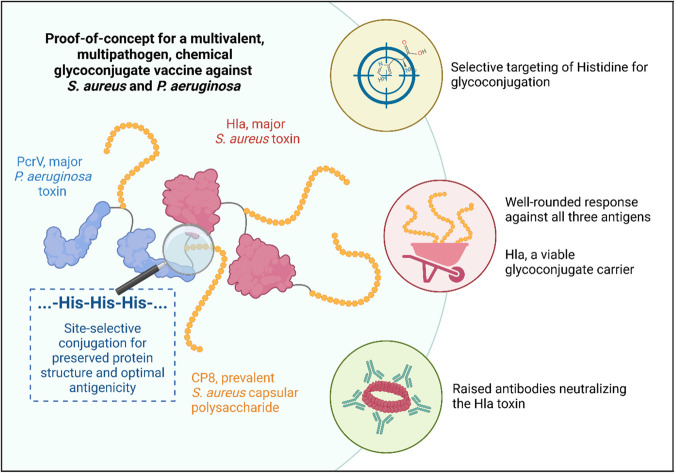

## Introduction

*Staphylococcus aureus* and *Pseudomonas aeruginosa* currently stand as two of the leading antimicrobial-resistant (AMR) pathogens, driving antibiotic drug inefficiency and persistent infections.^1–3^*P. aeruginosa* is an ubiquitous Gram-negative bacterium known for causing severe hospital-acquired infections^4^ with a mortality rate of 37%.^5,6^ It can lead to acute ventilator-associated pneumonia,^7,8^ is a known colonizer in chronic obstructive pulmonary disease and causes chronic infections in cystic fibrosis patients,^4^ leading to progressive lung decline.^9,10^ In the case of chronic wound infections, *P. aeruginosa* has been shown to synergize with other bacteria, primarily *S. aureus*.^11,12^*S. aureus* is a Gram-positive commensal bacterium, with a bacteremia mortality rate of about 18% in developed countries, and even higher in developing regions.^13^ It is the leading cause of nosocomial and community-acquired skin and soft tissue infections^14^ and surgical site infections.^15^ Both pathogens are classified as serious threats by the Centers for Disease Control and Prevention,^2^ and combating their infections remains a global challenge. However, despite decades of vaccine development, no effective preventive intervention is currently licensed for either bacterium.

Glycoconjugate vaccines offer a promising and safe approach to turn poorly immunogenic bacterial surface glycans into effective antigens by their covalent linkage to so-called protein carriers. These carriers provide epitopes capable of eliciting T cell help, thereby fostering a durable and buildable immune protection.^16–19^ Considering the complexity of bacterial pathogenesis and the multitude of virulence factors, as is the case for *S. aureus* and *P. aeruginosa*, a multiepitope approach, in which different antigens are simultaneously incorporated, is considered more promising for effective vaccines.^20^ Chemical glycoconjugation provides a prime opportunity for the coupling of several antigens, conveniently packed into a single construct for a potentially enhanced immunogenicity compared to monovalent formulations.^21^ In most of the licensed vaccines, the glycan antigens are incorporated in the carrier protein using a random conjugation procedure, which usually targets the most reactive amino acids, such as lysine or aspartic acid residues. However, this random approach may compromise some of the carrier's essential epitopes. In contrast, site-selective conjugation chemistry preserves critical protein epitopes, effectively promoting the protein carrier to a full-standing antigen.^22,23^ Two primary strategies for site-selective protein engineering in glycoconjugation are the integration of unnatural amino acids (uAA) and *in vivo* bioconjugation, also known as protein-glycan coupling technology.^16^ These approaches have gained significant interest in recent years and are currently undergoing clinical validation in several trials. Notably, a 9-valent bioconjugate vaccine against extraintestinal pathogenic *Escherichia coli* (ExPEC9V) for preventing invasive infection in older adults with a history of urinary tract infections has advanced to phase III (NCT04899336).^24^ Recent examples of glycoconjugates relying on uAA include Vaxcyte's 24- and 31-valent vaccines against *Streptococcus pneumoniae*, which were developed using a cell-free protein synthesis platform. Vaxcyte successfully completed phase II trials in adults for a 24-valent pneumococcal conjugate vaccine using this technology (NCT05266456),^25^ with ongoing validation studies in infants (NCT05844423). In all these vaccine candidates, which contained heterogeneous polysaccharides, site-selective approaches have been used to direct the conjugation to defined sites to preserve protein epitopes and improve product understanding.

Beyond the protein's dual role as both a carrier and antigen, the design flexibility of multipathogen glycoconjugate vaccines to target multiple bacteria^26^ is highly desirable, particularly against polymicrobial infections which are prevalent in nosocomial infections associated with AMR^27^ and for addressing synergistic interactions between pathogens.^11^ To develop carrier proteins that target multiple pathogens, novel carrier proteins can be designed to include the essential epitopes of various proteins from the target bacteria. In the bacteria's arsenal of virulence factors, *S. aureus* alpha-hemolysin (Hla) and *P. aeruginosa* V antigen (PcrV) stand out as potent cytotoxins that contribute significantly to their pathogenesis. Hla is a well-known pore-forming toxin that disrupts host cell membranes, leading to cell lysis and tissue damage.^28^ Neutralizing or attenuating its activity has shown promise in preclinical studies,^29,30^ and several vaccine candidates featuring Hla are in clinical trials.^13^ PcrV is the needle tip of the *P. aeruginosa* type III secretion system, a complex molecular machinery that injects bacterial effectors directly into host cells, promoting bacterial survival and dissemination.^31^ Recent research^32–35^ highlights the potential of PcrV as a target for vaccine development against *P. aeruginosa* infections. Anti-PcrV antibodies have also demonstrated protective effects in clinical settings.^36^

In this work, we present a novel strategy to generate a multivalent, multimeric vaccine targeting two different pathogens by generating fusion proteins, composed of several copies of Hla and PcrV, as a carrier for the site-selective conjugation of antigenic capsular polysaccharides (CP) of *S. aureus* serotype 8. CP8 from *S. aureus* is one of two serotypes associated with invasive disease and is known to play a crucial role in the pathogenesis of *S. aureus* infections by shielding the bacterium from host complement binding and phagocytic killing by neutrophils.^20^ CP8 has been used in vaccines that underwent clinical trials, such as Nabi's StaphVax and Pfizer's SA4Ag, both including a CP8 conjugate.^13^ Through a histidine-directed approach,^37^ Hla and PcrV were conjugated to sized CP8 fragments and assumed a dual carrier–antigen role. Notably, histidine-tagged proteins have been tested in the clinics and reported safe.^38,39^ The resulting multiepitope vaccine was compared in animal models to a classic CRM_197_-CP8 glycoconjugate benchmark.

## Results and discussion

### Design and production of multivalent, -meric and -pathogen PcrV/Hla protein carriers

**Fig. 1:**
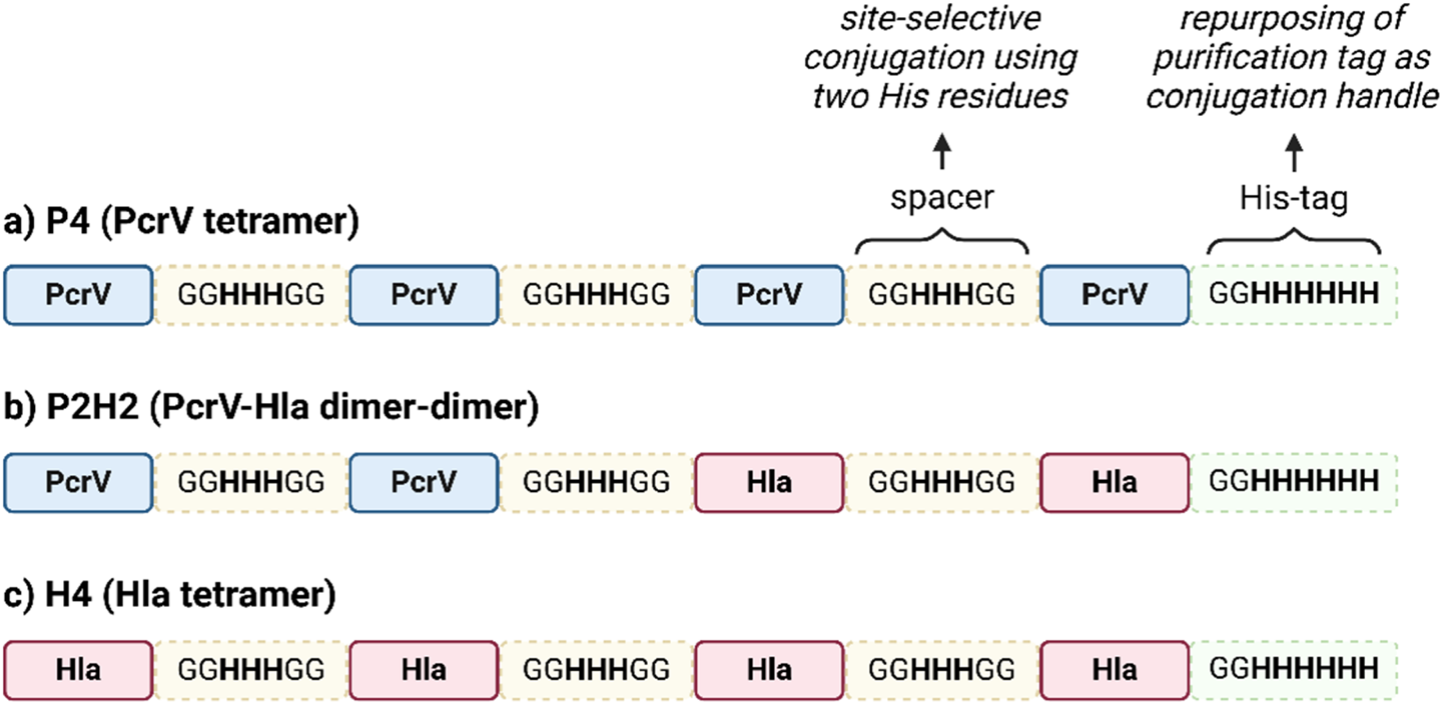
Schematic representation of the tetrameric fusion protein carriers with histidine (His_3_) spacers (in yellow) in between protein copies to serve as conjugation handles; a His_6_-tag (in green) is also present, both for purification and conjugation purposes; (a) PcrV fusion tetramer (P4), composed of 4 PcrV copies (in blue); (b) PcrV and Hla hybrid dimer–dimer with His_3_ spacers (P2H2); (c) Hla fusion tetramer (H4), composed of 4 Hla copies (in pink).

PcrV and Hla are both relatively small proteins, with a size around 32–33 kDa for the monomers.^28,40^ Protein size has been identified as an important feature for their carrier properties.^41^ Also, most of the carrier proteins exploited in licensed vaccines have a molecular weight ranging from 50 to 150 kDa.^23^ A design for a large construct, *i.e.* with several protein copies fused together, was therefore devised to increase the carrier size to provide a more concentrated source of protein epitopes while simultaneously combining both antigens, PcrV and Hla, into a single construct for a multivalent and multimeric approach against *P. aeruginosa* and *S. aureus* (Fig. 1).

All constructs were composed of chimeric tetramers of either PcrV (P4, Fig. 1a), Hla (H4, Fig. 1b), or featured a combination of the two (P2H2), in a consecutive dimer–dimer form (Fig. 1c). The sequences used for Hla correspond to the detoxified mutant, H35L (Hla_H35L_), as this point mutation prevents its natural toxic heptameric oligomerization.^28^ Native PcrV is also predicted to oligomerize into a pentamer;^42^ however, the recombinant wild-type (PcrV_wt_) was found to keep its monomeric nontoxic form. It was projected that inclusion of PcrV in the targeted multivalent fusion proteins would not be hampered by undesired oligomerization. To connect the protein monomers, several histidine-containing (His, H) spacers were introduced to separate protein copies and to serve as chemical conjugation points to the carbohydrate antigen. Peciak *et al.*^37^ developed a site-selective conjugation chemistry exploiting HxH motifs (where x can be any amino acid), *i.e.* on two histidine residues that are spatially close. The conjugation spacers used here thus contain a His_3_ sequence, flanked by glycine residues for flexibility. This conjugation chemistry also allowed to repurpose a His_6_-tag which was included in the construct to facilitate protein purification.

The summary of the tetramer production can be found in Table 1. PcrV was easily expressed in soluble form, while Hla_H35L_ showed a tendency to aggregate in inclusion bodies (Fig. S1†). As a result, the presence of Hla_H35L_ in the case of P2H2 reduced both the expression levels and the solubility of the tetramer. Sonication was found to be the most suitable lysis method as opposed to chemical lysis, as it enabled recovery of most proteins in soluble form.

**Table 1:** Summary of the purification process for P4, P2H2 and H4^a^

	P4	P2H2	H4
Purification step(s)	IMAC	IMAC (SEC)	IMAC, SEC
Purity	90%	81% (94%)	62%
Yield (per g of biomass)	7.2 mg	3.2 (2.0 mg)	0.7 mg

a Abbreviations: IMAC, immobilized metal-ion affinity chromatography; SEC, size-exclusion chromatography.

As the first purification step, all lysate supernatants were subjected to an immobilized metal-ion affinity chromatography (IMAC) binding the C-terminal His_6_-tag and the His_3_ spacers. In the case of P4, this first step was enough to yield a 90% purity, as determined through high performance liquid chromatography (HPLC) analysis (Fig. S2†). The high and effective full expression of PcrV compensated for the few PcrV-based impurities due to truncated forms of the full tetramer present after purification (Fig. S1†). However, in the case of P2H2, the presence of Hla reduced protein yields and introduced more impurities in the form of mono-, di- and trimers due to its less efficient expression. Therefore, a second purification step by size-exclusion chromatography (SEC) was needed, reaching 94% purity and decreasing the overall production yield. The third fusion protein, H4, gave the lowest yield and purity within the set of prepared tetramers. The H4 protein was also the least stable, requiring gentle manipulation as it was very prone to precipitation.

All chimeric protein tetramers were produced successfully, with the tetramer production comparable to that of the monomers. The expression of the hybrid PcrV and Hla dimer–dimer in particular (Fig. S1†) indicates that this strategy is suitable for multimeric fusion proteins. The P2H2 construct had production characteristics averaging those of the PcrV and Hla proteins, suggesting a certain level of predictability for multimeric constructs of protein antigens with established expression and purification protocols.

The tetramers were subjected to a Western Blot (WB) analysis to ascertain the conservation of continuous protein epitopes (Fig. S3†). As expected, both P4 and H4 were recognized by their corresponding antibodies, and P2H2 was recognized by both anti-PcrV monoclonal antibodies (mAb) and anti-Hla serum. While only P4 and P2H2 could be obtained with a purity degree equal or superior to 90%, the purity standard for manufacturing^23^ H4 was included in the study and used for the following conjugation.

### Generation and derivatization of CP8 oligosaccharides

**Fig. 2:**
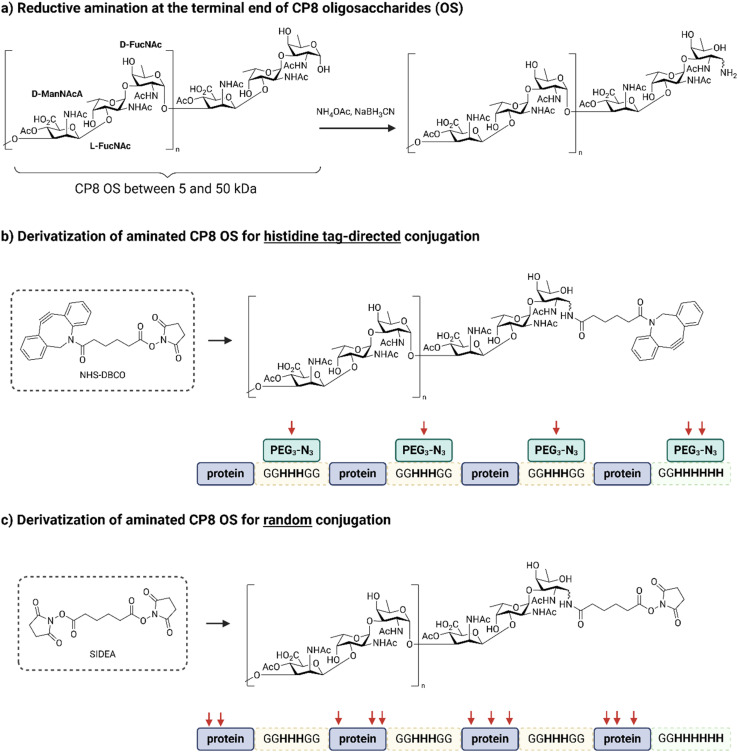
Generation of CP8 oligosaccharides (OS) and derivatization strategies for the His-tag-directed and random conjugation, with different click-chemistries and corresponding coupling points indicated with the red arrows; (a) after hydrolysis of the polysaccharide, the generated CP8 OS were sized and reductively aminated at their terminal end with ammonium acetate; (b) aminated CP8 OS were derivatized with NHS-DBCO for site-selective glycoconjugation onto the carrier protein derivatized beforehand with bis-sulfone-PEG_3_-N_3_ on HxH motifs; (c) aminated CP8 OS were derivatized with SIDEA (di-(*N*-succinimidyl)adipate) directly onto the lysine residue sidechains of the carrier protein, corresponding to a traditional random glycoconjugation.

**Fig. 3:**
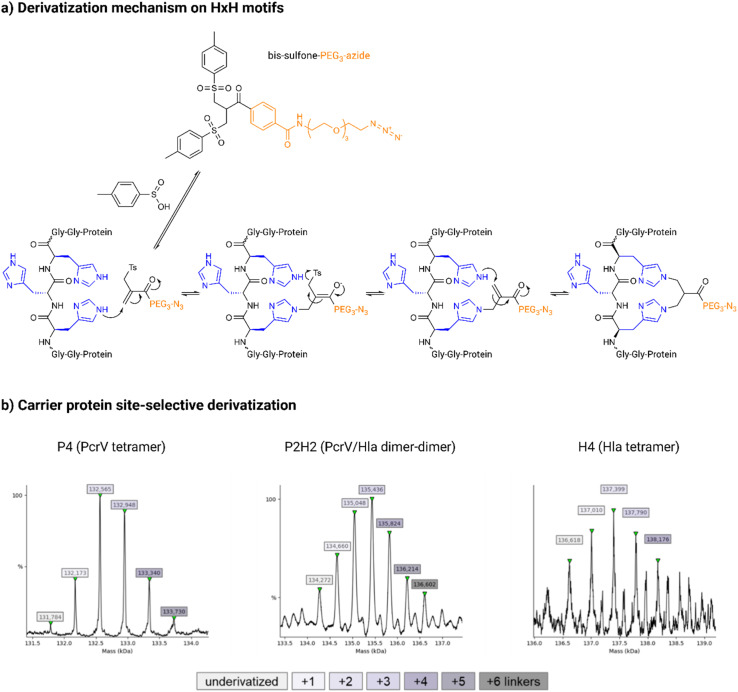
Site-selective derivatization of the carrier protein on HxH motifs (a) proposed derivatization mechanism: the linker is first equilibrated for 6 h at 37 °C in a 7 : 3 mixture of dimethyl sulfoxide (DMSO) and buffer (15 mM sodium phosphate, 1.5 mM ethylenediaminetetraacetic acid (EDTA), pH 5.0) to yield the reactive mono-sulfone after elimination of *p*-toluenesulfinic acid; the protein is then bis-alkylated at the imidazoles of spatially close histidine residues (in blue) by a sequence of addition–elimination reactions on the GGHHHGG (His_3_) spacers and the His_6_-tag inserted in the tetramers; (b) ESl-MS spectra from derivatized P4 (left), with 5 equivalents of bis-sulfone-PEG_3_-N_3_ linker, at 30% DMSO (underivatized peak at 131.784 kDa); P2H2 (center), with 8 equivalents of linker, without DMSO (underivatized peak at 134.272 kDa); H4 (right), with 10 equivalents of linker, without DMSO (underivatized peak at 136.618 kDa); all reactions were conducted at pH 5.0 in 50 mM sodium acetate for 25 h at room temperature (rt).

Conjugation of naturally sourced full-length polysaccharides is typically achieved in a random manner which can disrupt glycan epitopes as well as promote protein cross-linking and obstruction of key protein epitopes.^43^ This also prevents from obtaining a clear association between immunogenicity and the conjugation site. To obtain a more defined glycoconjugate structure and minimize these challenges, shorter and more homogeneous CP8 oligosaccharides (OS) were generated to be random and site-selectively conjugated through their terminal end to the fusion proteins (Fig. 2). To this end, CP8 was hydrolyzed in acetic acid obtaining OS fragments with an average weight of 25 kDa, resulting from a distribution ranging from 5 to 50 kDa (Fig. S4†). Aminated intermediates were then produced by reductive amination at the reducing end of the OS by treatment with ammonium acetate in the presence of sodium cyanoborohydride (Fig. 2). To compare the His-tag-directed and the classic random conjugation, the aminated CP8 OS were derivatized with different linkers (Fig. 2). For the preparation of the site-selective conjugates, a dibenzylcyclooctyne (DBCO) moiety was introduced to be coupled to the azide moiety of a PEG_3_-N_3_ linker.^37^ These were site-selectively attached to the histidine residues incorporated in the His_3_ spacers between the protein copies and the C-terminal His_6_-tag, through the use of a bis-sulfone reagent (Fig. 3). For the random conjugation strategy, the aminated OS were reacted with a bis(*N*-hydroxysuccinimide (NHS) ester) adipic acid linker, to obtain an active ester ready for the subsequent reaction with the primary amine in the sidechain of the protein lysine residues.

### Site-selective derivatization of the carrier proteins

For the site-selective glycoconjugates, the purified tetramers were first derivatized on the His_3_ spacers and His_6_-tag with a bis-sulfone-PEG_3_-N_3_ linker (Fig. 3). Bis-sulfone reagents can be used to bridge the imidazole rings of two nearby histidine residues in a protein by bis-alkylation (Fig. 3a). Bis-sulfones equilibrate to mono-sulfones, which can then engage in an addition–elimination mechanism relying on the Michael reaction and achieve a site-specific protein functionalization. Due to their lower p*K*_a_ compared to other nucleophilic residues, such as lysine and arginine, histidines remain unprotonated and reactive at mildly acidic pH (5–6), making derivatization of lysine residues less favorable under these conditions.^37^ Notably, this reaction can target native disulfide bonds in proteins,^44^ though these are absent in Hla and PcrV. Based on the design of the carrier proteins, it was expected that up to 6 linkers could be added to the HxH motifs, although derivatization of lysine residues and/or other spatially close histidine residues could result in the attachment of additional copies of the linker. Due to its phenyl groups, the bis-sulfone-PEG_3_-N_3_ is a poorly water-soluble compound and to solubilize a sufficient amount of the linker reagent, dimethyl sulfoxide (DMSO) was used as a cosolvent. The functionalized proteins were analyzed using electrospray ionization (ESI)-mass spectrometry (MS) to evaluate the derivatization level of the proteins (Fig. 3b), and nanodifferential scanning fluorimetry (nanoDSF) was used to exclude protein aggregation and loss of yield (Fig. S5†).

Incorporation of up to 5 linkers could be observed for P4 (133.730 kDa), with the majority of P4 carrying 2 or 3 linkers (132.565 and 132.948 kDa, respectively). For P2H2, the addition of up to 6 linkers (136.602 kDa) was detected; however, similarly to P4, an average incorporation of 2 and 3 molecules was achieved (135.048 and 135.436 kDa, respectively). Derivatization of H4 was slightly less effective, with MS analysis showing mostly the addition of only 2 linkers (137.399 kDa).

**Fig. 4:**
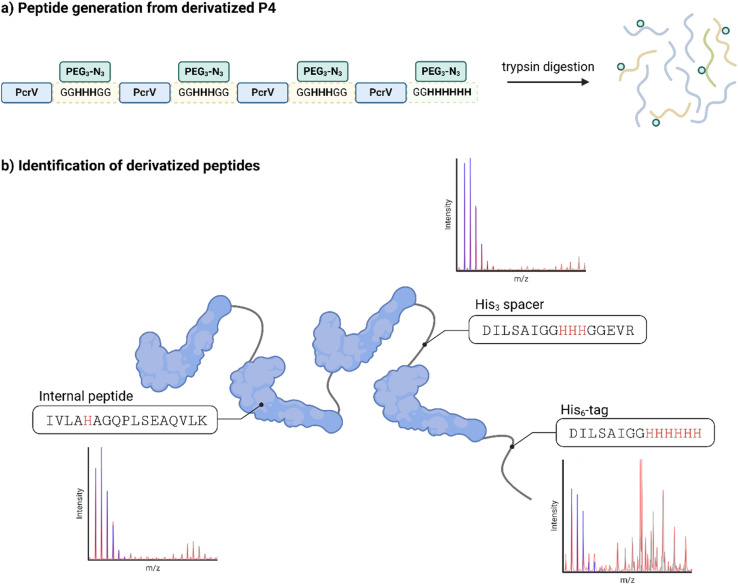
Schematic representation of the peptide mapping for a derivatized P4 sample to confirm the site-selectivity of the conjugation chemistry on histidine residues; (a) derivatized P4 was fragmented into peptides enzymatically by trypsin; (b) derivatized peptides were analyzed in ESI-MS against an underivatized reference (ESI, Fig. 6†) and three derivatization sites were confirmed.

The site-selectivity of the reaction was investigated through ESI-MS mapping of peptides derived from trypsin digestion of both underivatized and derivatized P4 samples (Fig. 4a). The selective digestion ensured the production of identical peptides from both derivatized and underivatized proteins, which minimized the risk of generating non-comparable peptides due to the derivatization process. Attached linkers could interfere with a random digestion (*e.g.* with pepsin) and complicate the peptide comparison. Addition of the azide linker (+388 Da) was observed in peptides with a MW corresponding to the His_3_ spacers, the His_6_-tag and to an internal PcrV peptide with one histidine and one lysine residue (Fig. 4b and S6†). While it was not possible to determine whether the linker in the latter peptide was attached through one or two arms of the linker (Fig. 3a), the linker was assumed to be connected through only the histidine residue, as trypsin cleaves at the C-terminus of lysine residues, and a derivatization at the side-chain would prevent cleavage after a functionalized residue.^45,46^ Notably, the C-terminal His_6_-tag peptide only carried one derivatization, and no double derivatization was identified despite the six consecutive histidine residues. While the generated peptides were not quantified, the three identified modifications can account for all the observed functionalization in the fusion proteins. There is, however, the possibility of other modifications, such as the derivatization of peptides with non-contiguous histidine residues, which would bridge two spatially close but distinct regions of the protein. This latter modification would not be detectable by the software using this technique, as it relies on the primary sequence of the protein. Notably, no peptides were found where lysine residues were functionalized with a linker.

The same analysis on P2H2 revealed similar results, with only traces of the functionalized internal peptide from PcrV (data not shown). The derivatization behaved therefore similarly with another protein, and no functionalized internal peptide was found in Hla, indicating that off-site functionalization, like that observed with the internal PcrV peptide, is likely a rare occurrence.

Taking together these data, all the modified proteins were primarily derivatized with a single linker at the purposely introduced histidine motifs, while modifications of other amino acid residues, such as lysine, were ruled out.

### Generation and immunization of the glycoconjugates

Once the site-selectivity of the derivatization of the carrier proteins on His_3_ spacers and His_6_-tag was confirmed, all conjugate vaccines were produced. Both site-selective and random conjugates with CP8 OS were generated for all tetramers, to compare the effect of the two conjugation chemistries on the immunogenicity of the conjugates. To evaluate the benefits of a tetrameric construct over a monomeric one, a random conjugate of a wild-type PcrV monomer (PcrV_wt_) to CP8 OS was also prepared. The final conjugates' production summary can be found in Table 2. The residual free protein and free saccharide were evaluated by sodium dodecyl sulfate-polyacrylamide gel electrophoresis (SDS-PAGE, Fig. S7†) and by HPLC, respectively, and the integrity of the proteins was assessed by WB (Fig. S8†). The random and site-selective conjugation chemistries resulted in considerable differences in saccharide loading (Table 2). Generally, random conjugates had a saccharide-to-protein weight ratio around 10 times higher than their site-selective counterparts, with the exception of P2H2, which showed similar ratios for both conjugation methods (0.7 and 0.9 w/w, respectively). Given the limited number of lysine residues in the protein monomers (16 and 28 for PcrV and Hla monomers, respectively), as well as the similar w/w ratios for the random PcrV-based conjugates and the random H4 conjugate, it was estimated that all the available lysine residues were most likely glycoconjugated in the random conjugates.

**Table 2:** High-performance anion-exchange chromatography with pulsed amperometric detection (HPAEC-PAD) analysis of the saccharide content of the CP8 conjugates generated with various protein carriers

		Saccharide/protein (w/w)
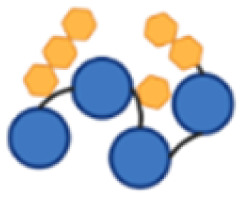	P4-CP8 site-selective	0.2
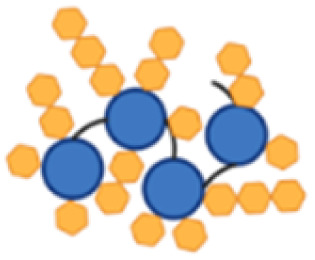	P4-CP8 random	2.2
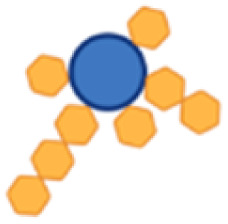	PcrV_wt_-CP8 random	4
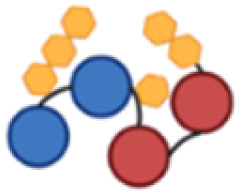	P2H2-CP8 site-selective	0.7
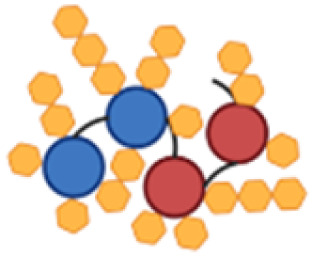	P2H2-CP8 random	0.9
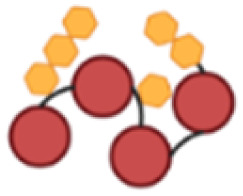	H4-CP8 site-selective	0.2
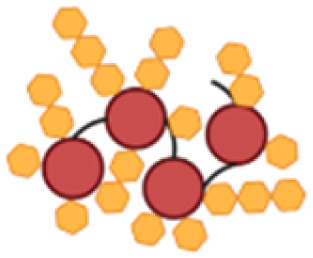	H4-CP8 random	1.9

To evaluate the vaccine potential of the new glycoconjugate *in vivo*, groups of eight CD1 mice were immunized on days 1 and 22 *via* intramuscular injection with 1 μg, based on saccharide content, of each multimeric construct. As controls, CP8 conjugates with monomeric protein PcrV_wt_ and the CRM_197_ carrier, commonly used in licensed vaccines, were used. Because the vaccine dosage was based on the saccharide content, resulting in varying amounts of proteins in the diverse glycoconjugates (Table S2†), two different dosages of the monomeric proteins PcrV_wt_ and Hla_H35L_ were chosen for comparison to closely match the protein amounts in the corresponding multimeric conjugates. Specifically, a dosage of approximately 5 μg was used to match the protein content of the P4 and H4 site-selective conjugates. For the P4, PcrV_wt_, and H4 random conjugates, as well as the random and His-directed P2H2 conjugates, the control protein dosage was set at 0.5 μg, reflecting the average protein content delivered by the conjugates.

**Fig. 5:**
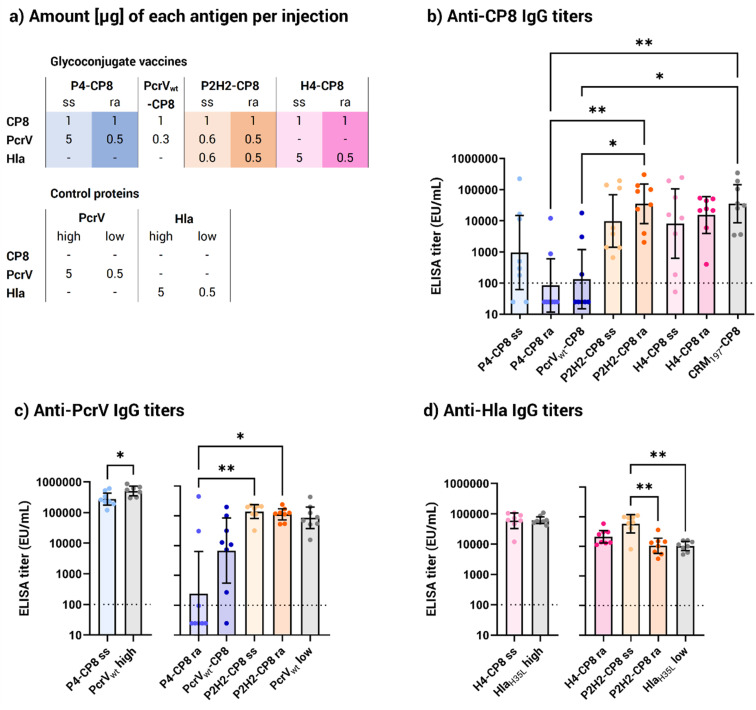
Immunological evaluation of the generated vaccines. (a) Summary of the injected amounts [μg] for each antigen included in the glycoconjugate vaccines. PcrV_wt_ and Hla_H35L_ were administered either as a 5 μg injection, equivalent to the average protein content in the site-selective P4 and H4 conjugates, or as a 0.5 μg injection, equivalent to the protein content in the random P4 and H4 conjugates and half of the P2H2 conjugate injections. Enzyme-linked immunosorbent assay (ELISA) immunoglobulin G (IgG) titers (b) anti-CP8, (c) anti-PcrV and (d) anti-Hla in mouse serum samples collected after 2 vaccine doses (EU mL^−1^, arbitrary units). For clarity, anti-PcrV and anti-Hla titers are shown alongside their corresponding control monomeric protein injection (in grey). Bars represent the geometric mean titers with 95% confidence intervals from 8 serum samples; p* < 0.05 and p** < 0.002 (Kruskal–Wallis and Dunn's test for multiple comparison, Mann–Whitney's test for single comparison). The dotted line at 100 EU mL^−1^ represents the detection limit of the assay, under which the titer is considered negative. Abbreviations: IgG, immunoglobulin G; ra, random conjugate; ss, site-selective conjugate.

All vaccines elicited an anti-CP8 response comparable to the CRM_197_ benchmark, except the P4 and PcrV_wt_ random conjugates (Fig. 5a). This consistent response across the conjugates was achieved despite varying CP8 OS loadings, including the site-selective conjugates having a lower saccharide content (Fig. 3b and Table 2). PcrV appeared as a weak carrier compared to Hla_H35L_ and CRM_197_, leading to lower anti-CP8 antibody levels. Both the site-selective and the random P4 conjugates failed to elicit a stronger immune response than the CRM_197_ control, indicating that neither the additional copies nor the multimeric presentation improved the carrier effect. However, the P4 site-selective conjugate showed a significantly stronger response than the P4 random conjugate, which gave a poor immune response, similar to the monomeric PcrV_wt_ random conjugate.

As opposed to the P4 conjugates, both the random and the site-selective P2H2 conjugates performed well in terms of anti-CP8 response, with significantly higher titers than P4. This suggests that the presence of the two Hla copies compensated for PcrV's limited ability as a protein carrier. Despite random conjugates generally carrying more saccharide moieties than their site-selective counterparts (Table 2), anti-carbohydrate immune responses were not statistically different between the random and the His-directed conjugates. In particular, the H4 site-selective conjugate showed a more scattered response than its random counterpart, although the difference was not statistically meaningful. Notably, the H4 site-selective conjugate had nearly 10 times lower saccharide loading than the H4 random conjugate (Table 2), suggesting that higher saccharide loading might help ensuring a more robust immune response. However, in the case of P2H2, where both random and His-directed conjugates bore a similar amount of glycan (0.9 and 0.7 w/w, respectively), the anti-CP8 antibody levels were clearly statistically comparable.

Overall, the site-selective conjugates achieved similar responses to the random ones, despite their lower carbohydrate loading (Fig. 3b), indicating that one attachment site was sufficient to provide a robust carbohydrate specific immune response with the CP8 OS length used.

Looking at the anti-PcrV responses, the P4 site-selective conjugate provided a more robust protein-specific response (Fig. 5b) compared to the P4 and PcrV_wt_ random conjugates, which yielded significantly lower titers. Two factors could explain this result. First, at the administered CP8 dose, the P4 site-selective conjugate had approximately 10 times more protein than its random counterpart. On the other hand, it should be considered that nearly all lysine residues of P4 and PcrV were engaged by the random conjugation, likely compromising the protein's integrity. Importantly, both the P2H2 random and site-selective conjugates elicited similar anti-protein antibody responses while having a similar glycan and protein content (∼0.5 μg at the tested CP8 dose), exceeding those achieved with the P4 random conjugate. On the whole, these findings suggest that PcrV alone is not an optimal carrier for glycoconjugate vaccines. However, fusing multiple copies of the same protein or combining it with Hla improves its carrier properties. In addition, directing the conjugation to specific sites can help preserve the protein structure and prevent the disruption of immunogenic protein epitopes.

Turning attention to the staphylococcal toxin, all Hla_H35L_-based conjugates elicited strong responses against Hla (Fig. 5c), comparable to those induced by the monomeric Hla_H35L_. As opposed to the anti-PcrV response, the P2H2 site-selective conjugate generated a noticeably superior response against Hla compared to the randomly conjugated version as well as the control Hla_H35L_. This improved response against Hla, which was not achieved by the H4 and P2H2 random conjugates of similar size, highlights the role of site-selective conjugation in enhancing the Hla-specific immune response, rather than the presence of a fused protein.

Combining the immune responses against PcrV, Hla and CP8, the P2H2 site-selective construct emerged as the lead vaccine candidate, generating a strong response against all three antigens. The strong responses elicited by the His-directed conjugates, particularly P2H2, against both protein antigens (Fig. 5b and c) highlighted the advantage of the site-selective chemistry for preserving critical protein epitopes, which is an important feature for proteins with the dual role of antigen and carrier. Furthermore, the fusion of Hla to PcrV was an impactful strategy to enhance the carrier properties of PcrV and achieved enhanced anti-CP8 immune responses compared to the PcrV monomer.

Next, the functional activity of anti-Hla antibodies was evaluated in a hemolysis inhibition assay, in which the capacity of the pooled mice sera to protect rabbit erythrocytes from the toxic activity of Hla wild-type (Hla_wt_) was assessed (Table 3). All sera obtained from two injections of the Hla-based conjugates demonstrated a neutralization capacity against Hla_wt_, equivalent to the controls.

**Table 3:** Neutralizing titers of the sera elicited towards the different conjugates measured against Hla_wt_^a^

		Sera neutralizing titers	
		Post 1	Post 2
Equivalence 1	H4-CP8 site-selective conjugate	80	1031
	Hla_H35L_ 5 μg	Negative	738
Equivalence 2	H4-CP8 random conjugate	Negative	605
	P2H2-CP8 site-selective conjugate	Negative	383
	P2H2-CP8 random conjugate	Negative	336
	Hla_H35L_ 0.5 μg	Negative	247

a For clarity, the titers were separated by comparable protein injections: Hla_H35L_ 5 μg is a monomeric protein injection equivalent to the average protein content for the site-selective H4 conjugate; Hla_H35L_ 0.5 μg is equivalent to the protein content for the random H4 conjugate and half of the P2H2 conjugate injections. The neutralization titer (IC50) is defined as the serum dilution giving 50% inhibition of hemolysis.

The site-selective and random P2H2 conjugates exhibited a neutralization titer of 383 and 336, respectively, which slightly outperformed the control protein (254), possibly due to the presence of two Hla copies, leading to different uptake and antigen processing, or to an adjuvant effect^19,47^ from the carbohydrate. Importantly, the H4 site-selective conjugate showed the highest neutralization titer in the set (1031) which was higher than its random counterpart (605). Also, the His-directed and random H4 conjugates gave a remarkably stronger functional response than the Hla_H35L_ monomer administered at the corresponding protein dosages (738 and 247, respectively). Given that H4 contains four copies of Hla_H35L_, this suggests that the number of protein copies, *i.e.* the multivalency of the construct, rather than the presence of CP8 is responsible for the increased antibody functionality. Additionally, the neutralizing titers of the site-selective conjugates generally exceeded those of their random counterparts, with the H4 site-selective conjugate showing a functional response (80) even after one dose (Fig. 5a and S9†). These data align with the measured antibody levels (Fig. 5c), corroborating the site-selective conjugation chemistry as a more suitable approach to preserve the Hla protein structure.

Altogether, the immune responses elicited by the PcrV- and Hla-based conjugates suggest that combining protein antigens can enhance the immune response against each respective antigen. These results support the overall hypothesis that carefully crafted conjugates can preserve critical epitopes, achieving a synergistic combination of protein and carbohydrate antigens.

## Conclusion

In this work, multimeric fusion proteins were used to compare a classic random conjugation strategy at lysine residues with the site-selective conjugation of spatially close histidine residues. Chimeric protein tetramers were successfully produced with yields comparable to monomers. A histidine-tag-directed derivatization proved to be a viable method for glycoconjugation, effectively preserving protein integrity and enhancing immune responses.

In terms of antibody levels, the P4 site-selective conjugate induced a stronger response than its random counterpart, likely due to the better preservation of protein epitopes. Most vaccines, except for the random PcrV-based conjugates, performed comparably to the CRM_197_ conjugate benchmark in eliciting a response against CP8. Notably, the histidine-tag-directed P2H2 conjugate showed a robust immune response against all three antigens, CP8, PcrV and Hla.

The use of fusion proteins to generate multivalent carrier proteins, paired with site-selective chemistry, shows promise for developing multipathogen vaccines. Fusion proteins performed as well as established carriers like CRM_197_, and in the case of P2H2, each antigen contributed to enhancing the overall immune response. The multivalency of chimeric proteins appears to offer an advantage over simply combining antigens into a single formulation, as indicated by the slight increase in antibody functionality observed with multivalent Hla carriers. Additionally, fusion proteins enable the synergistic combination of antigens, potentially boosting antibody titers, as suggested by the presence of PcrV enhancing the response against Hla in the P2H2 site-selective conjugate.

Histidine-directed conjugation emerges as a versatile method for coupling carbohydrates to proteins, offering advantages over other techniques such as bioconjugation in accommodating a wide range of saccharides, from synthetic oligosaccharides to full-length polysaccharides, and allowing fine control of the sugar-to-protein ratio. Although in the present work, achieving a high saccharide loading in the site-selective conjugates was not evaluated, incorporation of additional HxH motifs can be envisaged to increase the number of carbohydrate moieties attached.

Overall, the findings from this work on protein carrier design and conjugation strategies may be valuable in the development of future glycoconjugate vaccines aimed at targeting simultaneously multiple pathogens.

## Materials and methods

### Production and characterization of the chimeric protein carriers

All three designed plasmids corresponding to P4, P2H2 and H4 were synthesized by GeneArt (Thermo Fisher). The tetramer sequences were cloned into a pET303-CT vector including a His_6_-tag at the C-terminal.

P4 was expressed in BL21 Star (DE3) cells, while P2H2 and H4 were expressed in BL21 (DE3) cells (Fig. S1†). The cells were first grown at 37 °C for 8 h in lysogeny broth (LB) medium supplemented with 100 mg L^−1^ of ampicillin, and then transferred by a 1 : 100 dilution into HTMC medium (glycerol 15 g L^−1^, yeast extract 30 g L^−1^, MgSO_4_ 0.5 g L^−1^, KH_2_PO_4_ 5 g L^−1^, K_2_HPO_4_ 20 g L^−1^, KOH 1 M; pH 7.3) with a 24 h induction (1 mM IPTG) at 20 °C, 160 rpm. To improve H4 expression, induction trials at different timepoints were performed, and H4 expression was ultimately induced for 6 h at 20 °C, 160 rpm, to reduce the amount of Hla-based impurities. Cells were harvested and pelleted by centrifugation at 12 000 g, at 4 °C for 30 min. Cell pellets were resuspended in lysis buffer (30 mM Tris, pH 8.0, 0.25 mg mL^−1^ lysozyme, 1 mM MgCl_2_, 10 U benzonase, supplemented with EDTA-free complete Protease Inhibitor Cocktail; Roche) and lysed, either chemically for P4 through addition of the B-PER Bacterial Protein Extraction Reagent (Thermo Scientific), or mechanically through sonication for P2H2 and H4 (40% amplitude, with alternating pulse and rest cycles of 30 s on ice).

Lysis supernatants were collected by centrifugation at 12 000 g, at 4 °C for 30 min, and then purified by Co^2+^ affinity (HiTrap TALON crude; Cytiva) in a single-step elution in purification buffer (50 mM sodium phosphate, pH 8.0, 300 mM NaCl) with a gradient up to 250 mM imidazole. The collected and pooled fractions were concentrated in Phosphate Buffered Saline (PBS) buffer through Vivaspin® 15, 10 kDa MWCO, Centrifugal Concentrators (Sartorius). In the case of H4, the protein was subjected to an additional size-exclusion chromatography (SEC, Sephacryl S-200 HR; Cytiva) in PBS.

Protein concentrations were determined through bicinchoninic acid (BCA) assay against a bovine serum albumin (BSA) standard curve using the Pierce BCA Protein Assay Kit (Thermo Fisher).

The purified proteins were analyzed by SDS-PAGE and subsequently by WB (Fig. S3†). Gels were run using 4 to 12% Bis–Tris protein Gel NuPAGE, 4–12%, Bis–Tris, 1.0 mm, Mini Protein Gel, 12-well gels in morpholineethanesulfonic acid (MES) SDS running buffer (NuPAGE; Invitrogen). Proteins were transferred onto nitrocellulose membranes (0.2 μm pore size; NuPAGE; Invitrogen) for 7 min at 20–25 V using an iBlot module (Invitrogen). The membrane was blocked with 3% BSA in PBS overnight at 4 °C, then washed three times with PBS, supplemented with 0.05% Tween 20, and incubated for 1 h with anti-Hla serum or anti-PcrV mAb. The membrane was then washed again as described above and incubated for 1 hour with alkaline phosphatase (AP)-conjugated anti-mouse IgG (Sigma-Aldrich) diluted 1 : 1000 in blocking buffer. The membrane was finally washed three times and binding was detected using the colorimetric AP substrate reagent kit (Bio-Rad).

### Site-selective derivatization of the chimeric protein carriers

The bis-sulfone-PEG_3_-N_3_ linker (BroadPharm) was pre-incubated 6 h at 37 °C at a concentration of 2 mg mL^−1^, in a solution of 70% dimethyl sulfoxide (DMSO) and 30% buffer (15 mM sodium phosphate, 1.5 mM ethylenediaminetetraacetic acid (EDTA), pH 5.0) to yield the reactive mono-sulfone.

The protein derivatization reaction was carried out for 25 h at rt in 50 mM sodium acetate buffer at pH 5.0. The pH was an important parameter to keep below the p*K*_a_ of lysine residues, to ensure the selectivity on histidine. Different conditions for all tetramers were explored, reflecting their different behavior. P4 was incubated with 5 equivalents of linker in 30% DMSO, while P2H2 and H4 reacted with 8 and 10 equivalents of linker, respectively, in the absence of added DMSO. The derivatized proteins were purified twice through Zeba™ Spin Desalting Columns, 7 K MWCO, 0.5 mL (Thermo Scientific) in PBS.

The impact of the added 30% DMSO after 26 h was evaluated on P4 through nano differential scanning fluorimetry (Tycho, NanoTemper) and found to be acceptable (Fig. S5†).

### Generation and derivatization of the CP8 oligosaccharides

Isolated and purified CP8 was hydrolyzed in acetic acid 2% for 16 h, at 90 °C to OS of a MW ranging from 5 to 50 kDa, with an average of 35 kDa (depending on the CP8 batch, additional hydrolysis time was required, Fig. S4†). The reaction was neutralized with NaOH 2 M, the OS desalted in water through a 124 mL desalting column (Sephadex G-25; Cytiva) and lyophilized. The dry hydrolyzed OS were resuspended and sized through anion-exchange chromatography (HiTrap Q FF column 5 mL; Cytiva). Pools were analyzed by HPLC (TSK G3000PWXL; Tosoh Bioscience) against a range of pullulans, and merged for CP8 OS weighing between 5 and 50 kDa.

The sized CP8 OS were desalted again in water, lyophilized and resuspended in sodium acetate 150 mM, pH 7.0. The fragments were then aminated at their reducing end through reductive amination, with ammonium acetate (300 mg mL^−1^, added as a solid) and sodium cyanoborohydride (49 mg mL^−1^ solubilized in sodium acetate 150 mM, pH 7.0) at an approximate 3 mg mL^−1^ concentration of OS. The reaction was left for 4 days at 37 °C.

The aminated CP8 OS were first purified in NaCl 200 mM, and then desalted in water through a 124 mL desalting column (Sephadex G-25; Cytiva). Primary amines were quantified through colorimetric assay against a standard curve of 6-aminoacaproic acid. From there, two different OS derivatizations were performed depending on the conjugation strategy intended.

For the site-selective conjugation strategy, a dibenzylcyclooctyne (DBCO)-*N*-hydroxysuccinimide (NHS) ester linker was introduced at the aminated end of the OS, to be coupled to the azide (N_3_) moiety of a bis-sulfone-PEG_3_-N_3_ linker, site-selectively attached to the carrier protein beforehand. The aminated CP8 OS were solubilized in a 9 : 1 DMSO : H_2_O solution, to which 10 equiv. of linker and 5 equiv. of triethylamine were added. The reaction was left for 3 h at rt, desalted in water, lyophilized and analyzed by NMR to confirm the presence of DBCO.

For the random conjugation strategy, another bifunctional linker, the adipic acid bis(NHS ester), was inserted at the OS terminal amine, for subsequent reaction with the amine of the lysine residues' sidechains. The aminated CP8 OS were solubilized in a 9 : 1 DMSO : H_2_O solution, to which 10 equiv. of linker were added. The reaction was left for 2 h at rt, and purified by precipitation in 5 mL cold ethyl acetate, to which 250 μL of NaCl 3 M were added to favor OS precipitation. The solution was left for 1 h at 4 °C then centrifuged, the supernatant removed and the bottom phase washed with cold ethyl acetate. The OS were washed until a white precipitate was formed, which was lyophilized.

### Site-selectivity analysis by mass spectrometry

P4 and P2H2 were selectively digested and analyzed by ESI-MS in both their underivatized and derivatized forms with the bis-sulfone-PEG3-N3 linker. Trypsin (Sequencing Grade, modified from porcine pancreas; Serva), targeting arginine and lysine residues, was used for digestion. Protein samples were denatured at 95 °C for 5 minutes before digestion in PBS at a protein-to-trypsin ratio of 0.04 (w/w) for 3 hours at 37 °C.

Post-digestion, reversed-phase purification was performed using Oasis® HLB 30 μm sorbent cartridges (Waters). The cartridges were prepped per the manufacturer's instructions: 400 μL acetonitrile for activation, followed by equilibration with 600 μL of 0.1% aqueous formic acid thrice. Samples, acidified with 40 μL of 1% formic acid, were loaded and passed through the column multiple times for optimal peptide retention. The column was washed with 600 μL of 0.1% aqueous formic acid thrice to remove impurities. Peptides were eluted with 300 μL of 60% acetonitrile in 1% formic acid water twice, then concentrated and stored after solvent removal.

Peptides were injected at 200 μL min^−1^ in 100% buffer A (0.23% formic acid in water). Separation occurred through an ACQUITY UPLC^®^ HSS T3 reverse phase column (1.8 μm, 1.0 × 100 mm; Waters) with a gradient from 10% to 40% buffer B (0.23% formic acid in acetonitrile) over 6.8 minutes at 40 μL min^−1^.

The peptides were then infused into a SYNAPT G2-Si mass spectrometer (Waters) with a standard ESI source, calibrated with a 100 fmol μL^−1^ [Glu1]-fibrinopeptide solution. Data acquisition was performed in MSE resolution mode (300–2000 *m*/*z* range, 0.3 s scan time, 0.1 s interscan time, 4.0 eV trap collision energy, 2 eV transfer collision energy, 25 V cone voltage, 90 °C source temperature, 3000 V capillary voltage). Argon was used as the collision gas, with mass accuracy maintained by continuous Glu-Fib infusion at 5 μL min^−1^.

Peptides were identified using Protein Lynx Global Server 2.5 (Waters) and further verified with DynamX V 3.0 software (Waters). Selection criteria included peptides present in at least 4 of 5 replicates, <10 ppm mass error, and a minimum of 0.2 identified peptides per amino acid residue. Manual inspection was conducted to eliminate false identifications. Labeled peptides were analyzed and aligned using DynamX, followed by manual curation to ensure correct isotope alignment and assignment.

### Conjugation and characterization of the glycoconjugates

For site-selective conjugation, CP8 OS-DBCO samples were lyophilized and resuspended into concentrated derivatized tetramers in a theoretical 1 : 1 protein : OS ratio (as calculated with a theoretical OS concentration). For random conjugation, CP8 OS-SIDEA corresponding to 15 equivalents was weighed and resuspended into concentrated tetramers. The reactions were left overnight at 4 °C and purified through SEC (Sephacryl S-200 HR, Cytiva) in PBS for conjugates P4-CP8 random, P2H2-CP8 site-selective and random, P4-CP8 site-selective and random. The P4-CP8 site-selective conjugate was purified in PBS through ultracentrifugation (Vivaspin 0.5 mL, 50 K MWCO; Sartorius) at 4500 g (10 min cycles), however, protein recovery was suboptimal and SEC was chosen for the next conjugate. The conjugate was washed 30 times with 100 μL PBS1x. All conjugates were filtered sterile through 0.22 μm and the protein content was quantified by BCA assay.

Gels were run to confirm the minimal residual free protein using NuPAGE, 3–8%, Tris-acetate, 1.0 mm, Mini Protein Gel, 12-well gels in Tris-acetate SDS running buffer (NuPAGE; Invitrogen) (Fig. S7†).

The quantification of saccharide was performed by HPAEC-PAD analysis using standard samples of CP8 at seven increasing concentrations, ranging between 0.1 and 3.0 μg mL^−1^, to build the calibration curve. The reference and analytical samples were incubated at 100 °C for 3 hours in 4 M HCl, dried under vacuum (SpeedVac, Thermo), suspended in water and filtered with 0.45 μm Phenex-NY (Phenomenex) filters. The HPAEC-PAD analysis was performed in a Dionex ICS-6000 equipped with a CarboPac PA1 column (4 × 250 mm; Dionex) coupled with a PA1 guard to column (4 × 50 mm; Dionex) using isocratic elution in 16 mM NaOH.

Bacterial endotoxin levels were quantified through Limulus amebocyte lysate (LAL) assay using an Endosafe PTS spectrophotometer (Charles River) and found acceptable for immunization.

### Mice immunization and sera analysis

Animal studies were conducted according to experimental guidelines from the Animal Welfare Body of the Animal Resource Centre at GSK.

Groups of eight CD1 mice were immunized on days 1 and 22 by intramuscular injection of 1 μg (saccharide-based) of each glycoconjugate or 0.5/5 μg of monomeric control proteins, adsorbed on alum hydroxide adjuvant. Sera were bled at days 0 (pre-immunization), 21 (blood sample) and 42 (final bleed). IgG titers in collected sera were estimated by ELISA. 96-well microtiter plates were coated with 100 ng of CP8 polysaccharide. Plates were incubated overnight at 4 °C, washed three times with PBST (0.05% Tween-20 in PBS pH 7.4) and saturated with 250 μL per well of blocking buffer (3% bovine serum albumin in PBST) for 90 min at 37 °C. Two-fold serial dilutions of sera in blocking buffer were added to each well. Plates were then incubated at 37 °C for 1 h, washed with PBST, and then incubated for 90 min at 37 °C with anti-mouse (whole molecule) IgG-alkaline phosphatase (Sigma-Aldrich) diluted 1 : 2000 in blocking buffer. The plates were washed and developed with a 4 mg mL^−1^ solution of *p*-nitrophenyl phosphate (pNPP) in 1 M diethanolamine (DEA) pH 9.8, at rt for 30 min. The absorbance was measured using a SPECTRAmax plate reader 405 nm. IgG titers were calculated by the reciprocal serum dilution giving an Optical Density (OD) of 1.

Neutralization activity of anti-Hla antibodies was evaluated by hemolysis inhibition assay. Briefly, serial two-fold dilutions of serum (1 : 50 first dilution point) containing anti-Hla antibodies were incubated with native α-hemolysin (Hla_wt_) to allow the immunocomplex formation. Rabbit erythrocytes (RBC) were then added and lysed with residual free toxin. Hemolysis was evaluated by measuring the absorbance of the reaction supernatant at *A*_405nm_ which corresponds to the emission peak of hemoglobin. Raw data were analyzed through a SoftMaxPro template and the IC50 calculated by fitting a 4 Parameter Logistic (4 PL) regression curve. The neutralization titer (IC50) is defined as the serum dilution giving 50% inhibition of hemolysis.

## Data availability

The data supporting this work have been included as part of the article and the ESI.† Additional data can be made available on request.

## Author contributions

CS, JC, MRR, FC and RA conceived the study; CS, BM, DYW, BB, CG, GA, and GR performed experimental work; CS, JC, IM, MRR, FC and RA analyzed the results; CS, JC, FC and RA wrote the manuscript; all revised the manuscript.

## Conflicts of interest

BB, CG, GA, GR, IMR, MRR, FC and RA are employees of the GSK group of companies. CS and DYW participated in a post graduate studentship program at GSK. BM is a former GSK employee.

## Supplementary Material

SC-016-D4SC08622H-s001
