# Supplementary material for: Multimeric, multivalent fusion carrier proteins for site-selective glycoconjugate vaccines simultaneously targeting Staphylococcus aureus and Pseudomonas aeruginosa
Source: Chem Sci. 2025 Feb 20;16(13):5688–700. doi: 10.1039/d4sc08622h (PMC11875126; doi:10.1039/d4sc08622h)
Supplement: SC-016-D4SC08622H-s001 [file SC-016-D4SC08622H-s001.pdf]

Supplementary Information (SI) for Chemical Science.
This journal is © The Royal Society of Chemistry 2025

Supplementary Information

Multimeric, Multivalent Fusion Carrier Proteins for Site-Selective Glycoconjugate Vaccines Simultaneously Targeting Staphylococcus aureus and Pseudomonas aeruginosa

Charlotte Sorieul[a,b], Bartal Mikladal[b,c], Dung-Yeh Wu[a,b], Barbara Brogioni[b], Cinzia Giovani[b], Giusy Adamo[b], Giacomo Romagnoli[b], Immaculada Margarit Y Ros[b], Jeroen Codée[a], Maria R. Romano[b], Filippo Carboni*[b] and Roberto Adamo*[b]

[a] Leiden Institute of Chemistry, Leiden University, Einsteinweg 55, 2333 CC Leiden, Netherlands
[b] GSK Siena, Via Fiorentina, 1, 53100 Siena SI, Italy
[c] Current address: Genmab BV, Uppsalalaan 15, 3584 CT Utrecht, Netherlands
* Corresponding authors, e-mails: roberto.x.adamo@gsk.com and filippo.x.carboni@gsk.com

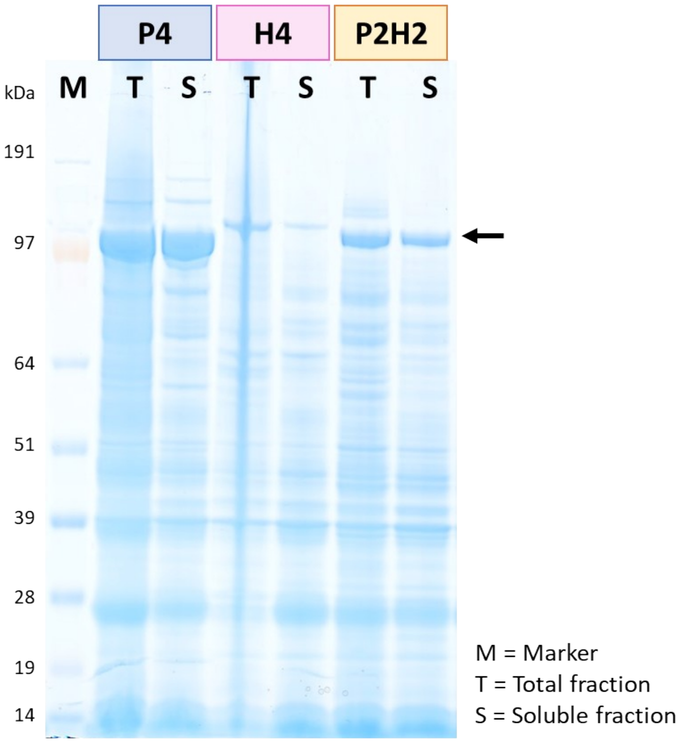

Figure S1. SDS-PAGE of *E. coli* cell lysates for all tetramers after a 24 h induction with isopropyl β-D-1-thiogalactopyranoside (IPTG). The arrow indicates bands corresponding to the tetramers, at the expected Mw around 132 kDa for P4, 137 kDa for H4 and 134 kDa for P2H2. After lysis, a sample of the total fraction (T) was taken to evaluate the amount of protein expressed in inclusion bodies. After centrifugation, a sample of the lysate supernatant (S) was collected to evaluate the soluble protein.

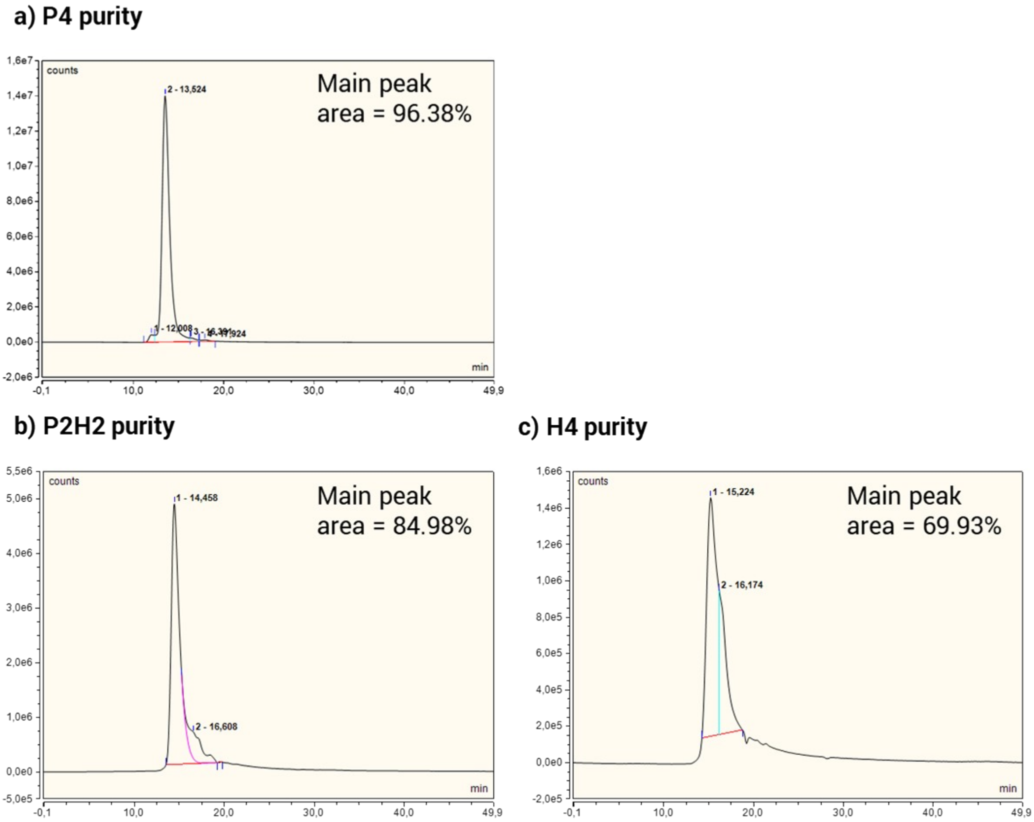

Figure S2. Purity analysis of the purified tetramers **a)** P4, **b)** P2H2 and **c)** H4. Spectra from UV/Vis 214, 230, 254, 260 and 280 nm were analyzed, peaks were identified and average purity calculated at all mentioned wavelengths to give values reported in **Table 1**. Only the spectra corresponding to excitation/emission 280/330 nm are here shown. HPLC analysis run on a TSK3000PWXL column in NaPi 100 mM, Na_2SO_4 100 mM, Acn 5%, pH 7.2.

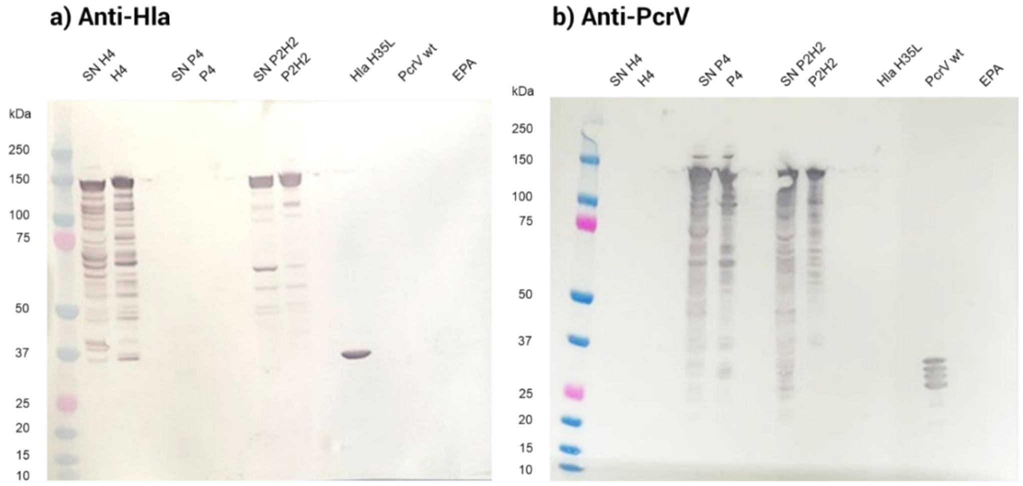

Figure S3. Western Blot against **a)** Hla (anti-Hla serum) and **b)** PcrV (anti-PcrV mAb) for all produced tetramers. Samples of the lysis supernatant (SN) was run alongside purified samples. Monomeric proteins Hla_{H35L} and $PcrV_{wt}$ were added as positive controls. Exoprotein A (EPA) from *P. aeruginosa* was added as negative control.

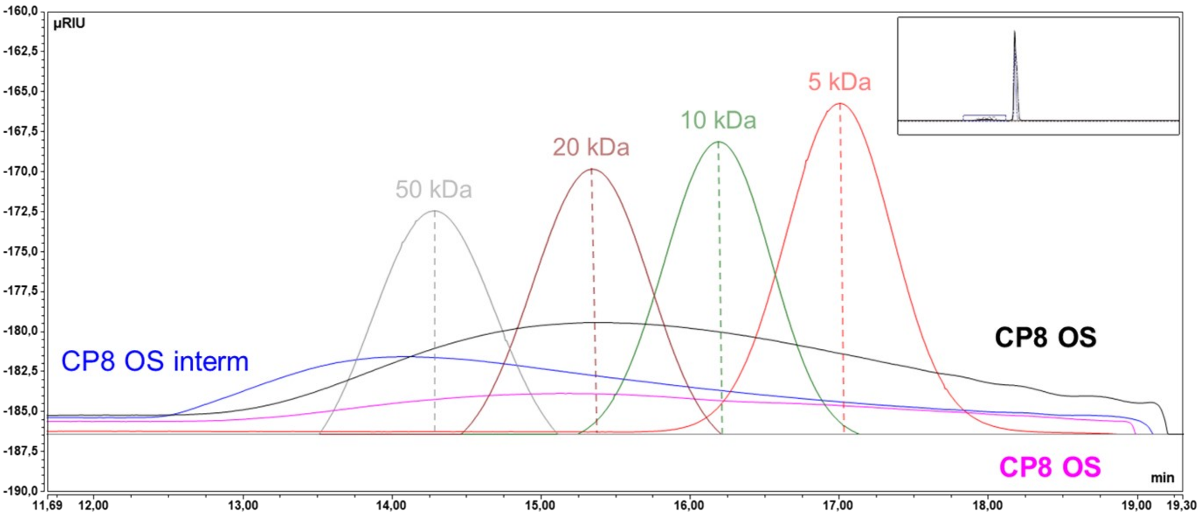

CP8 OS batch used for site-selective conjugation

CP8 OS batch used for random conjugation

Figure S4. HPLC chromatograms in infrared of CP8 OS post hydrolysis against a range of pullulans (50 kDa in grey, 20 kDa in brown, 10 kDa in green and 5 kDa in red). The first batch (in pink) was used mainly for the site-selective conjugation, while the second one (in black) was used for the random conjugation. Both batches show a final similar trend, although the second one required an additional 8 h of hydrolysis from the initial 16 h (in blue) to match the first obtained OS. Analysis was run on a TSK3000PWXL column in NaPi 100 mM, Na_2SO_4 100 mM, Acn 5%, pH 7.2; an average of 30 kDa was estimated for the batch in pink, 20 kDa for the batch in black.

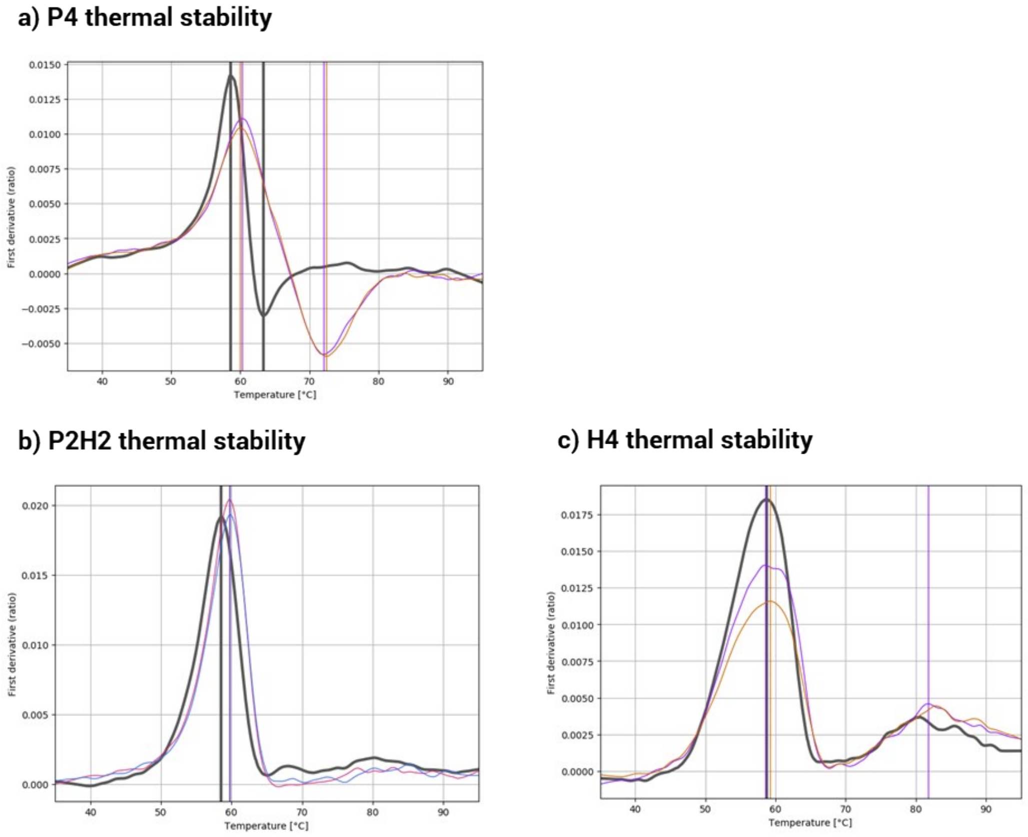

Figure S5. Thermal stability analysis of a) P4, b) P2H2 and c) H4 after a 26 h incubation in 30% DMSO. Reference in PBS (in grey), incubation at pH 5.0 (in purple) and pH 8.0 (in red).

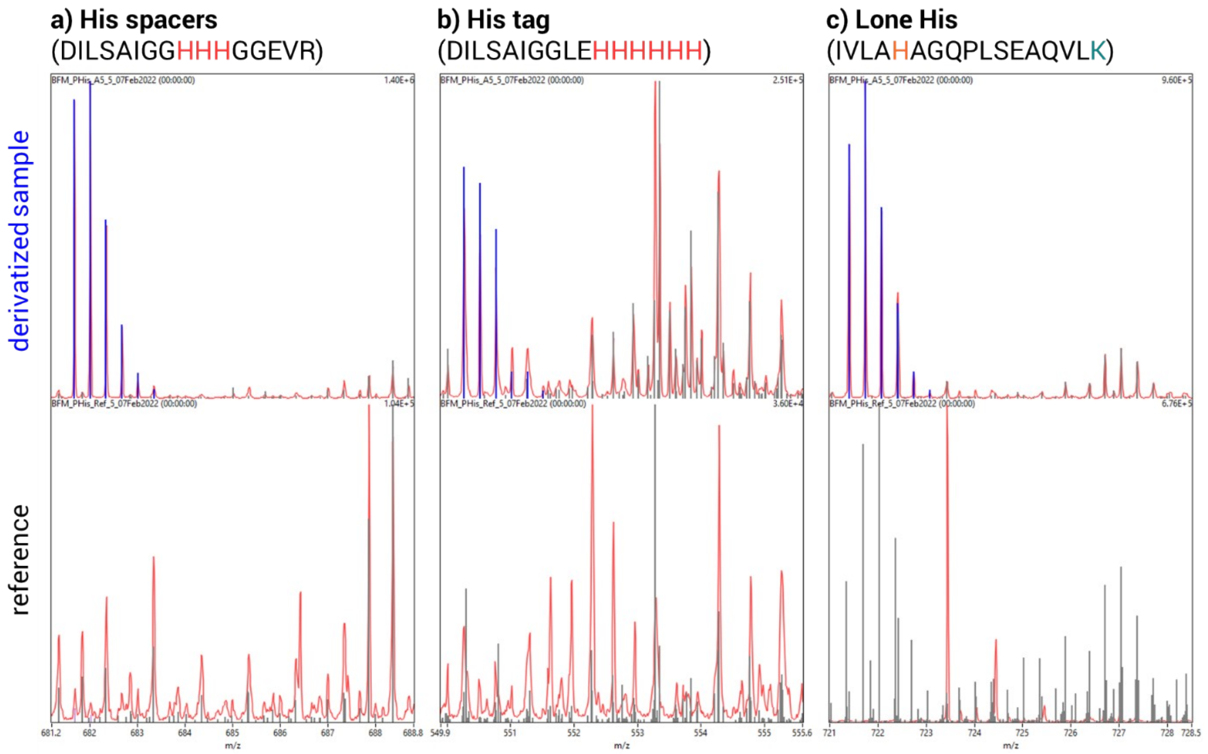

Figure S6. ESI-MS analysis of site-selectivity with derivatization hits (+388 Da, corresponding to the addition of one linker) **a)** on His_3 spacers, **b)** on His_6-tag and **c)** on a single histidine-containing peptide. The top panels show the presence of the linker (in blue) in the P4 derivatized sample, with several ionization states. The lower panels show the reference sample, for the underivatized P4 sample. Derivatized peptides were identified with the DynamX software (Waters).

Table S1. Purification description of the CP8 glycoconjugates. Single-step purifications might indicate the presence of free saccharide, accounting for some of the observed saccharide content (**Table 2**).

Abbreviations: SEC, size-exclusion chromatography.

	**Purification method**
**P4-CP8 site-selective**	Ultrafiltration
**P4-CP8 random**	SEC (x2)
**PcrV-CP8 random**	Precipitation $(NH_4)_2SO_4$
**P2H2-CP8 site-selective**	SEC (x2)
**P2H2-CP8 random**	SEC
**P4-CP8 site-selective**	SEC
**P4-CP8 random**	SEC

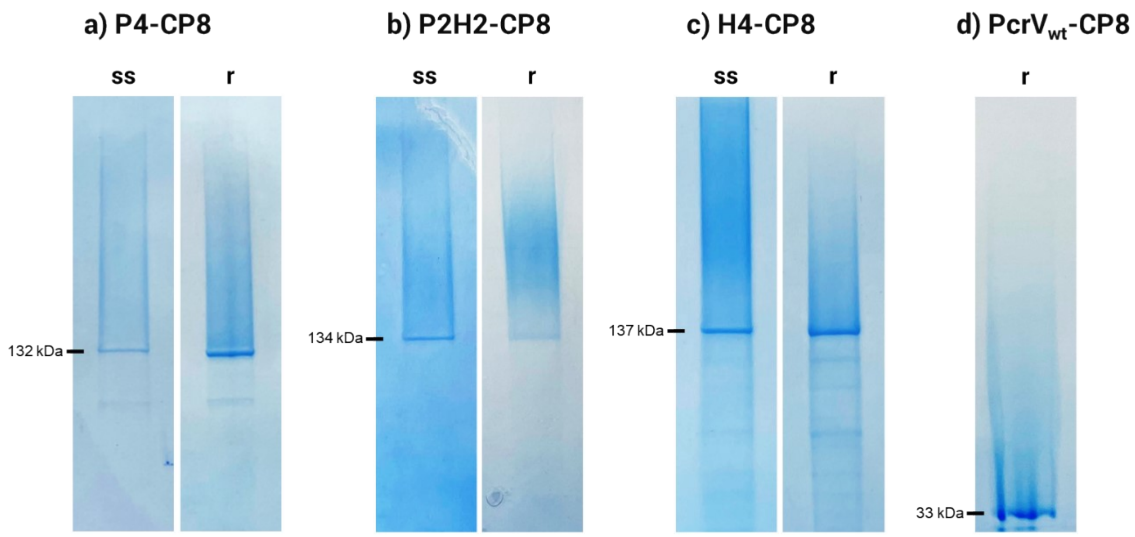

Figure S7. SDS-PAGE of purified conjugates, both site-selective (ss) and random, **a)** P4-CP8, with residual unconjugated P4 at 132 kDa, **b)** P2H2-CP8, with residual unconjugated P2H2 at 134 kDa, **c)** H4-CP8, with residual unconjugated H4 at 137 kDa and **d)** random-only $PcrV_{wt}$-CP8, with residual unconjugated $PcrV_{wt}$ at 33 kDa.

Abbreviations: r, random conjugate; ss, site-selective conjugate.

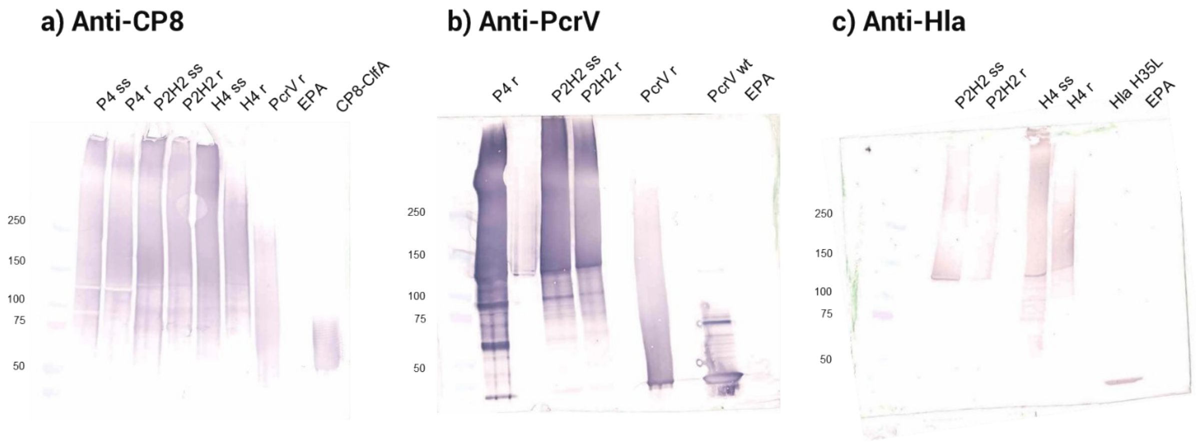

Figure S8. WB of the CP8 glycoconjugates, with antibody response against all three antigens, CP8, PcrV and Hla.

Abbreviations: ClfA, clumping factor A (from *S. aureus*); EPA, exoprotein A (from *P. aeruginosa*); r, random conjugate; ss, site-selective conjugate.

Table S2. Summary of the injected amounts of saccharide and protein (in µg) per injection, per mouse, for each conjugate vaccine and protein control.

Immunization	Dose saccharide [µg]	Dose protein [µg]
**P4-CP8 site-selective**	1	4.57
**P4-CP8 random**	1	0.45
**PcrV-CP8 random**	1	0.25
**P2H2-CP8 site-selective**	1	1.27
**P2H2-CP8 random**	1	1.07
**H4-CP8 site-selective**	1	4.93
**H4-CP8 random**	1	0.54
**PcrV wt** (equivalent to the average of P4-CP8 random, PcrV-CP8 random and half P2H2-CP8 site-selective and random)	-	0.43
**PcrV wt** (equivalent to P4-CP8 site-selective)	-	4.57
**Hla H35L** (equivalent to the average H4-CP8 random and half P2H2-CP8 site-selective and random)	-	0.57
**Hla H35L** (equivalent to H4-CP8 site-selective)	-	4.93
**CRM_{197}-CP8**	1	-

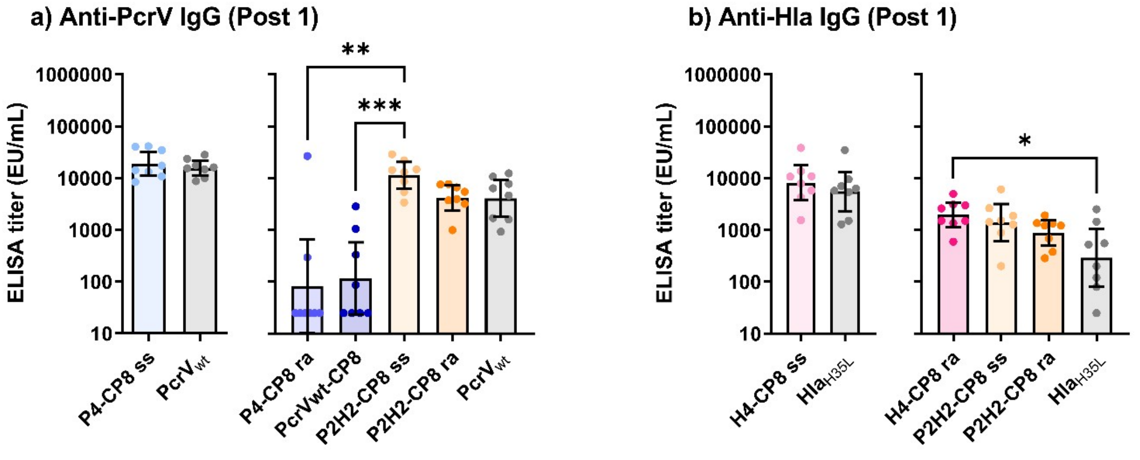

Figure S9. Enzyme-linked immunosorbent assay (ELISA) immunoglobulin G (IgG) titers **a)** anti-PcrV and **b)** anti-Hla in mouse serum samples collected after 1 vaccine dose (EU/mL, arbitrary units); bars represent the geometric mean titers with 95% confidence intervals from 8 serum samples; p* < 0.05, p** < 0.002, p*** < 0.0002 (Kruskal–Wallis and Dunn's for multiple comparison, Mann-Whitney's test for single comparison).

Abbreviations: IgG, immunoglobulin G; ra, random conjugate; ss, site-selective conjugate.

Table S3. Protein sequences used for the generation of the P4, P2H2 and H4 tetramers featuring the His-containing motifs for glycoconjugation. The sequences were cloned into the pET303-CT plasmid including a His_6-tag at the C-terminal.

P4 (4 copies of PcrV monomers)

```
MEVRNLNAARELFLDELLAASAAPASAEQEELLALLRSERIVLAHAGQPLSEAQVLKALAWLLAANPSAPP
GQGLEVLREVLQARRQPGAQWDLREFLVSAYFSLHGRLDEDVIGVYKDVLQTQDGKRKALLDELKALTAEL
KVYSVIQSQINAALSAKQGIRIDAGGIDLVDPTLYGYAVGDPRWKDSPEYALLSNLDTFSGKLSIKDFLSG
SPKQSGELKGLSDEYPFEKDNNPVGNFATTVSDRSRPLNDKVNEKTTLLNDTSSRYNSAVEALNRFIQKYD
SVLRDILSAIGGHHHGGEVRNLNAARELFLDELLAASAAPASAEQEELLALLRSERIVLAHAGQPLSEAQV
LKALAWLLAANPSAPPGQGLEVLREVLQARRQPGAQWDLREFLVSAYFSLHGRLDEDVIGVYKDVLQTQDG
KRKALLDELKALTAELKVYSVIQSQINAALSAKQGIRIDAGGIDLVDPTLYGYAVGDPRWKDSPEYALLSN
LDTFSGKLSIKDFLSGSPKQSGELKGLSDEYPFEKDNNPVGNFATTVSDRSRPLNDKVNEKTTLLNDTSSR
YNSAVEALNRFIQKYDSVLRDILSAIGGHHHGGEVRNLNAARELFLDELLAASAAPASAEQEELLALLRSE
RIVLAHAGQPLSEAQVLKALAWLLAANPSAPPGQGLEVLREVLQARRQPGAQWDLREFLVSAYFSLHGRLD
EDVIGVYKDVLQTQDGKRKALLDELKALTAELKVYSVIQSQINAALSAKQGIRIDAGGIDLVDPTLYGYAV
GDPRWKDSPEYALLSNLDTFSGKLSIKDFLSGSPKQSGELKGLSDEYPFEKDNNPVGNFATTVSDRSRPLN
DKVNEKTTLLNDTSSRYNSAVEALNRFIQKYDSVLRDILSAIGGHHHGGEVRNLNAARELFLDELLAASAA
PASAEQEELLALLRSERIVLAHAGQPLSEAQVLKALAWLLAANPSAPPGQGLEVLREVLQARRQPGAQWDL
REFLVSAYFSLHGRLDEDVIGVYKDVLQTQDGKRKALLDELKALTAELKVYSVIQSQINAALSAKQGIRID
AGGIDLVDPTLYGYAVGDPRWKDSPEYALLSNLDTFSGKLSIKDFLSGSPKQSGELKGLSDEYPFEKDNNP
VGNFATTVSDRSRPLNDKVNEKTTLLNDTSSRYNSAVEALNRFIQKYDSVLRDILSAIGGLEHHHHHH
```

P2H2 (2 copies of PcrV, followed by 2 copies of Hla monomers)

```
MEVRNLNAARELFLDELLAASAAPASAEQEELLALLRSERIVLAHAGQPLSEAQVLKALAWLLAANPSAPP
GQGLEVLREVLQARRQPGAQWDLREFLVSAYFSLHGRLDEDVIGVYKDVLQTQDGKRKALLDELKALTAEL
KVYSVIQSQINAALSAKQGIRIDAGGIDLVDPTLYGYAVGDPRWKDSPEYALLSNLDTFSGKLSIKDFLSG
SPKQSGELKGLSDEYPFEKDNNPVGNFATTVSDRSRPLNDKVNEKTTLLNDTSSRYNSAVEALNRFIQKYD
SVLRDILSAIGGHHHGGEVRNLNAARELFLDELLAASAAPASAEQEELLALLRSERIVLAHAGQPLSEAQV
LKALAWLLAANPSAPPGQGLEVLREVLQARRQPGAQWDLREFLVSAYFSLHGRLDEDVIGVYKDVLQTQDG
KRKALLDELKALTAELKVYSVIQSQINAALSAKQGIRIDAGGIDLVDPTLYGYAVGDPRWKDSPEYALLSN
LDTFSGKLSIKDFLSGSPKQSGELKGLSDEYPFEKDNNPVGNFATTVSDRSRPLNDKVNEKTTLLNDTSSR
YNSAVEALNRFIQKYDSVLRDILSAIGGHHHGGASADSDINIKTGTTDIGSNTTVKTGDLVTYDKENGMLK
KVFYSFIDDKNHNKKLLVIRTKGTIAGQYRVYSEEGANKSGLAWPSAFKVQLQLPDNEVAQISDYYPRNSI
DTKEYMSTLTYGFNGNVTGDDTGKIGGLIGANVSIGHTLKYVQPDFKTILESPTDKKVGWKVIFNNMVNQN
WGPYDRDSWNPVYGNQLFMKTRNGSMKAADNFLDPNKASSLLSSGFSPDFATVITMDRKASKQQTNIDVIY
ERVRDDYQLHWTSTNWKGTNTKDKWIDRSSERYKIDWEKEEMTNGGHHHGGASADSDINIKTGTTDIGSNT
TVKTGDLVTYDKENGMLKKVFYSFIDDKNHNKKLLVIRTKGTIAGQYRVYSEEGANKSGLAWPSAFKVQLQ
LPDNEVAQISDYYPRNSIDTKEYMSTLTYGFNGNVTGDDTGKIGGLIGANVSIGHTLKYVQPDFKTILESP
TDKKVGWKVIFNNMVNQNWGPYDRDSWNPVYGNQLFMKTRNGSMKAADNFLDPNKASSLLSSGFSPDFATV
ITMDRKASKQQTNIDVIYERVRDDYQLHWTSTNWKGTNTKDKWIDRSSERYKIDWEKEEMTNGGLEHHHHH
H
```

H4 (4 copies of Hla monomers)

```
MASADSDINIKTGTTDIGSNTTVKTGDLVTYDKENGMLKKVFYSFIDDKNHNKKLLVIRTKGTIAGQYRVY
SEEGANKSGLAWPSAFKVQLQLPDNEVAQISDYYPRNSIDTKEYMSTLTYGFNGNVTGDDTGKIGGLIGAN
VSIGHTLKYVQPDFKTILESPTDKKVGWKVIFNNMVNQNWGPYDRDSWNPVYGNQLFMKTRNGSMKAADNF
LDPNKASSLLSSGFSPDFATVITMDRKASKQQTNIDVIYERVRDDYQLHWTSTNWKGTNTKDKWIDRSSER
YKIDWEKEEMTNGGHHHGGASADSDINIKTGTTDIGSNTTVKTGDLVTYDKENGMLKKVFYSFIDDKNHNK
KLLVIRTKGTIAGQYRVYSEEGANKSGLAWPSAFKVQLQLPDNEVAQISDYYPRNSIDTKEYMSTLTYGFN
GNVTGDDTGKIGGLIGANVSIGHTLKYVQPDFKTILESPTDKKVGWKVIFNNMVNQNWGPYDRDSWNPVYG
NQLFMKTRNGSMKAADNFLDPNKASSLLSSGFSPDFATVITMDRKASKQQTNIDVIYERVRDDYQLHWTST
NWKGTNTKDKWIDRSSERYKIDWEKEEMTNGGHHHGGASADSDINIKTGTTDIGSNTTVKTGDLVTYDKEN
GMLKKVFYSFIDDKNHNKKLLVIRTKGTIAGQYRVYSEEGANKSGLAWPSAFKVQLQLPDNEVAQISDYYP
RNSIDTKEYMSTLTYGFNGNVTGDDTGKIGGLIGANVSIGHTLKYVQPDFKTILESPTDKKVGWKVIFNNM
```

VNQNWGPYDRDSWNPVYGNQLFMKTRNGSMKAADNFLDPNKASSLLSSGFSPDFATVITMDRKASKQQTNI
DVIYERVRDDYQLHWTSTNWKGTNTKDKWIDRSSERYKIDWEKEEMTNGGHHHGGASADSDINIKTGTTDI
GSNTTVKTGDLVTYDKENGMLKKVFYSFIDDKNHNKKLLVIRTKGTIAGQYRVYSEEGANKSGLAWPSAFK
VQLQLPDNEVAQISDYYPRNSIDTKEYMSTLTYGFNGNVTGDDTGKIGGLIGANVSIGHTLKYVQPDFKTI
LESPTDKKVGWKVIFNNMVNQNWGPYDRDSWNPVYGNQLFMKTRNGSMKAADNFLDPNKASSLLSSGFSPD
FATVITMDRKASKQQTNIDVIYERVRDDYQLHWTSTNWKGTNTKDKWIDRSSERYKIDWEKEEMTNGGLEH
HHHHH
